# Children and Young Adults with Epilepsy Exhibit an Interictal Autonomic Dysfunction: A Prospective Exploratory Study

**DOI:** 10.3390/brainsci14070670

**Published:** 2024-06-29

**Authors:** Carmen Salluce, Marco Cocciante, Marisa Gazzillo, Anna Rita Ferrari, Roberta Battini, Filippo Maria Santorelli, Emanuele Bartolini

**Affiliations:** 1 Department of Developmental Neuroscience, IRCCS Stella Maris Foundation, 56128 Pisa, Italy; carmen.salluce@fsm.unipi.it (C.S.); marco.cocciante@fsm.unipi.it (M.C.); m.gazzillo@santobonopausilipon.it (M.G.); annarita.ferrari@fsm.unipi.it (A.R.F.); roberta.battini@fsm.unipi.it (R.B.); filippo.santorelli@fsm.unipi.it (F.M.S.); 2 Department of Clinical and Experimental Medicine, University of Pisa, 56126 Pisa, Italy; 3 Division of Pediatric Neurology, Department of Neurosciences, Santobono-Pausillipon Children’s Hospital, 80129 Naples, Italy; 4 Tuscany PhD Program in Neurosciences, 50139 Florence, Italy

**Keywords:** electrodermal skin conductivity, childhood epilepsy, dysautonomia, pediatric

## Abstract

Dysautonomic disorders are an increasingly studied group of conditions, either as isolated diseases or associated with other neurological disorders. There is growing interest in understanding how dysautonomia affects people with epilepsy, who may report autonomic symptoms before, during and after seizures. Furthermore, autonomic abnormalities appear to play a role in sudden unexpected death in epilepsy, likely contributing to the increased mortality rate described in epilepsy. To better understand the association between epilepsy and dysautonomia, we explored electrochemical skin conductance in a group of 18 children and young adults with epilepsy compared to 15 age- and sex-matched healthy controls by the Sudoscan^TM^ test. We found a significant difference in terms of electrochemical skin conductance, suggesting that people with epilepsy suffer significantly reduced conductance in small nerve fibers. Within patients, values were significantly different according to the type of epilepsy and to neuroimaging results, with lower conductance values in epilepsies of unknown origin and in patients with morphological abnormalities of the brain. Using a non-invasive test, we identified altered conductance of small sympathetic nerve fibers in children and young adults with epilepsy, suggesting underlying dysautonomia. Further studies are needed to investigate this association and to clarify its neurobiological substrates.

## 1. Introduction

The autonomic nervous system (ANS) regulates the function of almost all tissues in the body, providing innervation to smooth muscle, cardiac muscle and pacemaker cells, exocrine and endocrine glands, white and brown adipose tissue, liver cells and lymphatic tissue [1]. The ANS is typically divided into three parts: the sympathetic, parasympathetic and enteric systems. Although this division can be useful for understanding the anatomy of this system, it can be inaccurate when considering function, as the different parts of the ANS are integrated to ensure fine regulation of the innervated organs, working antagonistically or synergistically [2]. Due to the wide distribution of the ANS, dysautonomias are characterized by a large number of symptoms (e.g., impaired thermoregulation, respiration, heart rate, blood pressure and sleep disturbances), which can either result from a dysfunction of the central autonomic network (CAN) or from the involvement of autonomic peripheral fibers [3,4,5,6]. Autonomic disorders are well studied in the adult population, both in their primary etiologies and in their secondary causes; the growing interest in these disorders is partly due to the impact they have in the daily lives of patients with well-known chronic disorders such as Parkinson’s disease, Lewy body dementia, pure autonomic failure, diabetes mellitus, Guillain-Barré syndrome, etc. [7]. Dysautonomias are less studied in the pediatric population, but there is a growing interest in deepening the knowledge of these conditions [5,8,9] in order to better understand and diagnose them and find appropriate treatments.

In children, dysautonomia can be overt in genetic disorders, especially yielding defects in neurotransmission (e.g., Dopamine-hydroxylase deficiency), mitochondrial or lysosomal function (e.g., Leigh syndrome, Fabry disease) but it has also been observed in premature children or in those with Autism Spectrum Disorders [5]. Indeed, symptoms can be subtle and overlooked in pediatric neurological disorders, as attention might be drawn to the main symptoms of the disease that do not include self-evident dysautonomic symptoms. One particular condition in which dysautonomia seems to play an important role is epilepsy. ANS involvement in children with epilepsy can be seen at several levels. However, it is an under-recognized aspect in the diagnosis and management of epilepsy. Autonomic symptoms may manifest as the direct clinical expression of an epileptogenic zone or the secondary involvement of the CAN after spreading of epileptiform discharges. A few pediatric epilepsy syndromes, such as Self-Limited Epilepsy with Autonomic Seizures (SeLeAS) have prominent autonomic manifestations during seizures, which helps in the diagnosis and possible subsequent selection of anti-seizure medications (ASMs) [10]. ASN dysfunction can also be a comorbidity in a child with epilepsy secondary to a genetic, metabolic or mitochondrial disorder. ASN dysfunction is almost always found in generalized tonic-clonic seizures and prolonged status epilepticus and may also occur in focal seizures.

ASN dysfunction is also implicated in patients with SUDEP. In addition, ASMs can cause side effects that may involve the autonomic nervous system. Finally, ASN involvement may also have therapeutic implications, as in the case of vagus nerve stimulation (VNS) devices, which repeatedly stimulate the vagus nerve and thus the autonomic network, treatment approved by the Food and Drug Administration (FDA) for refractory epilepsy. Recognition of autonomic nervous system involvement, therefore, can contribute to the effective management of children with epilepsy [11]. There are several testing modalities to assess dysautonomia in PWE; some of the most common are electrocardiogram recordings, heart rate variability (HRV), electrodermal activity, oxygen saturation, EEG video monitoring and magnetic resonance imaging (MRI) [12]. These methods can be performed by assessing interictal, peri-ictal, ictal or post-ictal ANS activity.

The aim of our study was to investigate ANS functioning in a cohort of pediatric and young adult patients with epilepsy compared to a group of healthy controls by means of a non-invasive investigation, such as the electrochemical skin conductance test using the Sudoscan^TM^ technique. All subjects with epilepsy were assessed in the interictal period, and the possible association between skin electrochemical conductance values and the clinical features of PWE was investigated.

## 2. Materials and Methods

The study was systematically proposed to patients on follow-up at our specialized center for epilepsy, based upon the following inclusion criteria: (i) diagnosis of epilepsy, according to the definition of the International League Against Epilepsy [13]; (ii) age = 5–24-years-old; (iii) preserved language comprehension; (iv) ability to maintain a still position, according to clinician judgement; (v) patient and parental consent. Three patients refused to perform the exam and five were excluded due to movement artefacts during the assessment. We enrolled and analyzed eighteen consecutive patients (Table 1) (mean age 14.3 years; SD = 5.6) compared to fifteen age and sex-matched healthy controls (mean age 11.6 years; SD = 3.3) (*p* > 0.05). All patients had preliminarily been studied by video-EEG recording (10–20 International System); neuroimaging was performed in all those with focal epilepsy by 1.5 or 3 T MRI. The classification of seizure and epilepsy type was performed by two experienced clinicians (E.B. and A.R.F.). The Sudoscan^TM^ testing was performed during the daytime, in full wakefulness. The Sudoscan^TM^ device calculates electrodermal skin conductivity (ESC) measured in microsiemens (μS) in response to a low-voltage electric current (<4 V) generated on steel plates in contact with the skin of the palms of the hands and soles of the feet. The higher the value obtained for each limb, the more intact are the small sympathetic cholinergic fibers that stimulate sweat secretion in response to the stimulus. The test took about three minutes and required a fair degree of compliance from the patient, who must remain stationary, standing or sitting, with palms and feet adhered to the surface of the machine’s plates during acquisition. The test measured the conductance in left- and right-hand and foot [14,15]. We analyzed the mean ESC resulting from all the measures as well as the EZSCAN score. Sudoscan^TM^ testing and the EZSCAN score in particular have been developed to investigate small autonomic neuropathies in diabetes mellitus or glucose intolerance [16]. The EZSCAN score, calculated from hands and feet ESC values, relies on comparison between hand and feet patterns and demographic data (age and BMI). No subject in the control group took any medication. All individuals performed the exam in the morning at least three hours after their last meal. None had performed physical activity beforehand. All subjects and/or caregivers provided written informed consent and the study procedures complied with the Declaration of Helsinki.

We performed the statistical analysis using the R studio software package (Version: 2024.04.0+735). Binary parameters were tested by the χ^2^ test for categorical variables. Comparisons of the quantitative variables between groups were performed by parametric tests (independent samples *t*-tests and one-way ANOVAs), after correlation analysis (Supplementary Materials).

## 3. Results

According to the type of seizures we classified the epilepsy of the study population as focal (*n* = 5), generalized epilepsy (*n* = 11), combined generalized (*n* = 1) and unknown (*n* = 1). Etiology was genetic in a single patient carrying a pathogenetic variant in *SLC6A1* (#9) and structural in two patients (#8; #15; Table 1), while for the others the etiology remains unknown or is being further investigated. Most patients had generalized (*n* = 11/18) or focal epilepsy (*n* = 5); combined epilepsy and unknown classification were applied to one patient each. Most patients took ASMs at the time of testing (*n* = 11/18), mostly valproate as monotherapy (*n* = 9) or polytherapy (*n* = 2), except one patient who was on CBZ monotherapy [Table 1]. Preliminarily, we tested whether age at testing and sex could correlate with ESC and EZSCAN scores. We found no correlation, either in patients or in controls.

We then explored the differences between groups and found statistically significant differences for both mean ESC (t(31) = 3.56, *p* = 0.001, Cohen’s d = 1.24) and EZSCAN scores (t(31) = −4.10, *p* < 0.001, Cohen’s d = −1.43). Specifically, patients exhibited lower mean ESC (65.00 vs. 79.11 μS) and higher EZSCAN scores (13.27 vs. 4.60), indicating overall lower conductivity. Focusing on the group of patients, we did not identify significant correlations between ESC, age at epilepsy onset or duration of active epilepsy.

To assess the differences in conductivity according to epilepsy characteristics we performed one-way ANOVA testing, alternatively using mean conductivity and EZSCAN score as the dependent variables and epilepsy type, seizure frequency and cognitive status as independent variables. We found a statistically significant effect of epilepsy type on the mean ESC (F(3,14) =7.87, *p* = 0.002), with higher values for focal epilepsies (73.18 μS, SD = 12.48) compared to generalized epilepsies (65.08 μS, SD = 6.03). The single subject with combined epilepsy had low values (65.87) and the patient with unknown etiology was indeed an outlier (22.35 μS).

Similarly, patients with generalized epilepsies had higher EZSCAN scores (13.45, SD = 5.59) than those with focal (11.20, SD = 4.79), combined (3) and unknown (32) epilepsies.

Conversely, we did not identify statistically significant differences according to the seizure frequency (*p* = 0.69 for mean conductivity and *p* = 0.48 for EZSCAN scores) and to the cognitive status (*p* = 0.83 for mean conductivity and *p* = 0.98 for EZSCAN scores).

We also performed an independent samples *t*-test according to treatment status and neuroimaging features to verify whether these parameters could affect conductance. We found no statistically significant differences for ongoing ASMs (*p* = 0.67 for mean ESC and *p* = 0.88 for EZSCAN scores). The only patient taking CBZ (Patient 14) was not an outlier compared to those on therapeutic regimens including VPA. The conductivity tended to be higher in patients with normal neuroimaging compared to those with abnormal MRIs (mean values = 69 vs. 53 μS), although with no statistical significance (*p* = 0.06). Accordingly, EZSCAN scores tended to be lower in PWE with normal neuroimaging (mean values = 11.15 vs. 17,75, *p* = 0.12).

## 4. Discussion

The involvement of the autonomic nervous system in children and young adults with epilepsy is still poorly recognized, both from the diagnostic and therapeutic standpoints. Berg et al. found the presence of dysautonomic symptoms in children with epileptic and developmental encephalopathies (DEEs) by means of questionnaires administered to parents, showing the correlation between dysautonomia, the extent of the child’s functional impairment and increased caregiver stress [17]. Our study similarly showed significant involvement of the ANS through altered conductance at the level of the small sympathetic fibers that regulate sweating. It is known that PWE may suffer a disturbance of autonomic functions in the peri-ictal phase induced by seizures themselves (palpitations, breathing difficulties, nausea, vomiting, gustatory sensations, sphincter incontinence, flushing and changes in pupil diameter). Dysautonomic symptoms are almost always found in tonic-clonic seizures and in prolonged status epilepticus and may also occur in focal seizures. Dysfunction of the ANS also seems to be implicated in the pathogenetic mechanism of Sudden Unexpected Death in Epilepsy (SUDEP) [11], which to date is still not fully understood.

The hypotheses linking epilepsy to the presence of dysautonomic disorders are to be found in the ‘central’ portion of the ANS, the central autonomic network (CAN). This circuit, consisting of the amygdala, anterior cingulate gyrus, insula, thalamus, hypothalamus, periaqueductal grey, parabrachial nucleus and other regions of the brainstem, presides over the regulation of the peripheral portion of the ANS [5,18]. Epileptic seizures can influence the function of the ANS directly by activating autonomic pathways and indirectly through reflex responses to the behavioral aspects of the seizure and the release of catecholamines by the adrenal gland [18]. It has been hypothesized that CAN activation during tonic-clonic seizures plays a role in autonomic manifestations in individuals who develop SUDEP [12]. The MORTEMUS study [19] showed that in cases of SUDEP after generalized tonic-clonic seizures recorded on video-EEG, there is a common cardio-respiratory pattern characterized by a short period of normal or increased heart and respiratory rate, after which central apnea, severe bradycardia and often transient asystole occur together with post-ictal generalized EEG suppression (PGES) on EEG. This sequence is terminal in one-third of cases, while in the remainder it is followed by a phase of improvement and subsequent respiratory deterioration with further apnea and asystole.

This phenomenon is probably multifactorial and there is considerable evidence of the role of genetic factors. There are some genetically based epilepsies in which the risk of SUDEP appears to be increased compared to epilepsy in general. This is the case for individuals with pathogenic variants in genes such as *SCN1A*, *SCN8A* and *SCN2A*. It has been hypothesized that the reason for the increased risk of SUDEP is related to the fact that the protein products of these genes are localized both at the Central Nervous System level and at the cardiac level possibly favoring proarrhythmogenic states or bradycardia [20].

For example, in channelopathies such as Dravet syndrome (associated with a *SCN1A* mutation), autonomic and cardiac dysfunction has been hypothesized during paroxysmal episodes in fever. It has been shown that in such patients, even in the interictal period, there is an alteration at the autonomic level, with a predominance of sympathetic tone with reduced HRV values compared to healthy controls and other epileptic syndromes [21].

Epilepsy could also be associated with the alteration of other autonomic functions, including the regulation of blood pressure, temperature and sweating, as well as gastrointestinal and/or urinary functions.

Recently, Marchal and Rheims, in their review, discussed the data available in the literature on the clinical features and probable pathophysiology of epilepsy-related non-cardiac and non-respiratory autonomic dysfunctions, as well as the various objective tests available to assess them [22].

The authors emphasized that altered sweat gland activity, which can be assessed by various methods, including the measurement of electrodermal activity (EDA), is more frequent than other autonomic manifestations in epilepsy. Labuz-Roszak and Pierzchala showed that abnormal EDA values are found in about one-third of patients during interictal states, whereas cardiovascular changes due to autonomic dysfunction are less common [23]. EDA, which measures changes in skin electrical conductance due to pure sympathetic activity, may therefore be an interesting biomarker when other indices remain normal [24].

A review by Casanovas Ortega et al. reported an increase in EDA in the peri-ictal phase, in both generalized and focal seizures [24]. According to some studies, the augmentation of sympathetic activity recorded by EDA correlates with increased duration of PGES following generalized seizures in both adults and children [25,26]. An increased duration of PGES (especially greater than 50 s) would also correlate with an increased risk of SUDEP [27]. Poh et al. have also shown that during PGES there is sympathetic activation with increased post-ictal EDA and concomitant parasympathetic suppression evidenced on electrocardiography by high-frequency power suppression on HRV assessment [28].

There is also evidence that EDA may also increase in the pre-ictal period as a possible predictor of seizures [24,26].

With regard to the interictal period, the scenario is likely to be different. Horinouchi et al. explored interictal electrodermal activity by using a wristband in adult PWE and found reduced values compared to healthy controls, with wider differences for patients suffering many seizures [29]. However, autonomic functions measured by wearable devices may show only minor differences, likely due to the limited sensibility of the methods. Indeed, Vieluf et al. also used multi-day wristband data that did not reliably distinguish the interictal from the peri-ictal phase in terms of EDA [30]. Instead, Drake et al. demonstrated altered sympathetic functioning in adults by means of sympathetic skin responses, which had higher values and longer latencies in patients compared to healthy subjects [31]. Koseoglu et al. measured autonomic functions by using heart rate variation during resting activity, heart rate variation in response to deep breathing and blood pressure response to rising quickly from the supine position; they found that hippocampal sclerosis and a duration of epilepsy longer than 10 years were associated with parasympathetic dysfunction, while seizure repetition showed sympathetic autonomic dysfunction [32].

Sivathamboo and Perucca have recently reviewed all the studies published up till 2021, ascertaining that PWE often exhibit autonomic changes during the interictal period in terms of HRV, EDA and Baroreflex sensitivity, especially those with temporal lobe epilepsy, Dravet syndrome, drug-resistant and chronic forms [33].

The technique we employed has never been used in epilepsy. The study of adrenergic function was conducted with the assessment of sudomotor function by measuring electroclinical skin conductance. Currently, sudomotor function can also be assessed by Quantitative Sudomotor Axon Reflex Testing, Thermoregulatory Sweat Testing, Sympathetic Skin Response or skin biopsy. However, these tests remain scarcely used, especially in the pediatric population, due to the need for training for the researcher, the time needed for the tests or their invasive nature. We employed the Sudoscan^TM^ system (Impeto Medical, Paris, France), which has recently been proposed as a standardized, easy and painless tool for the assessment of sudomotor function by the measurement of skin electrochemical conductance (ESC). Unlike the Sympathetic Skin Response, it directly assesses sweat gland function: the device uses very low-intensity direct current stimulation and reverse ionophoresis to measure local conductance derived from the electrochemical reaction between sweat chloride ions and nickel included in stainless steel electrodes in contact with the palms of the hands and soles of the feet [34,35]. In addition, normative data on the pediatric population are available. ESC values depend on sweat gland density, which has been shown to be equivalent in infants and adults, explaining why ESC values observed in children are comparable to those in adults [36].

In our study, we found a reduction in skin conductance in children and young adults with epilepsy compared to healthy controls, demonstrating an interictal impairment of the sympathetic autonomic system. We may hypothesize that measuring electrochemical conductance by Sudoscan^TM^ can catch differences that would pass unnoticed using wearable devices.

Several clinical studies and experimental models have suggested that autonomic dysregulation is induced by repeated seizures and epileptic discharges and is not present at the onset of epilepsy; specifically, autonomic centers would be continuously stimulated or inhibited by repeated seizures and interictal epileptic activity, raising the risk of SUDEP, especially in drug-resistant PWE [37,38]. However, the liaison between recurring drug-resistant seizures and autonomic dysfunctions has been assessed almost exclusively in adults by cardiovascular testing. In this regard, Liu et al. demonstrated that multiple, long-duration seizures were associated with cardiac autonomic dysfunction [39]. In Dutsch et al.’s 2004 study [40] this hypothesis was confirmed by a decrease in cardiovascular sympathetic activity after temporal lobe epilepsy surgery.

Strikingly, other studies have shown no differences in sympathetic activity between individuals with well-controlled and intractable temporal lobe epilepsy using heart rate variability [41], suggesting that seizure repetition is not the only mechanism of ANS dysfunction.

In our analysis, in fact, we found no statistically significant correlations according to age of onset, duration of active disease, frequency of seizures and current therapy intake. In relation to this last aspect, the respective roles of epilepsy and ASM on autonomic functions remain a matter of debate. Some studies have reported that an autonomic dysfunction involving both sympathetic and parasympathetic activities can be associated with the use of voltage-dependent sodium channel blockers, including phenytoin, carbamazepine or oxcarbazepine [42,43]. On the other hand, other studies have not found a significant association between ASM and autonomic disorders [44]. Of note, a single study performed in children with epilepsy found that abrupt ASM discontinuation can cause an unbalance in cardiovascular autonomic functions [42]. On the other hand, Said Berilgen et al. found an improvement in sympathetic activity measured by SSR and RR interval after ASM introduction in focal epilepsies [45]. In our cohort, driven by a large prevalence of valproate intake, we found no difference with respect to patients off-therapy, suggesting that autonomic dysfunction can be attributable to the epilepsy rather than the drug regimen. However, the duration of therapy intake in our cohort was short, and we cannot rule out that longer therapeutic regimens might influence the ANS. A potential effect of valproate on autonomic balance has been demonstrated in prenatal autism model mice [46], yet no evidence is available in vivo.

We found a statistically significant effect according to the type of epilepsy. Patients with generalized epilepsy exhibited lower conductance values than those with focal forms. Generalized epilepsies may be characterized by a widely distributed disruption of brain networks that hypothetically are more likely to influence CAN function. Conversely, in focal epilepsies, the effects of seizure type and interictal activity or brain lesions on the ANS may be different and autonomic changes may vary depending on the lateralization of epileptic foci [38]. Furthermore, we observed that conductivity tended to be better in patients with normal brain imaging results. In our population, abnormal MRI findings varied from arachnoid cysts to ventricular dysmorphisms or porencephaly due to perinatal suffering. Associations between white matter abnormalities and cardiac dysautonomic symptoms are described in the literature [47]. Indeed, volumetric alterations in the gray matter of regions in areas involved in cardiorespiratory regulation (amygdala and parahippocampal gyri) have been described in focal forms [48], especially in temporal lobe epilepsy [49]. Moreover, recent findings suggest that the function of the thalamus crucially supports the CAN and this subcortical nucleus has shown functional and structural defects in different types of generalized epilepsies [50,51,52].

## 5. Conclusions

Our work showed that children and young adults with epilepsy, irrespective of type, etiology, treatment with ASM and duration of illness, exhibit an aberrant skin electrical conductance, pointing to an underlying autonomic dysfunction, compared to healthy controls. The concordance of the result with other previously performed studies makes the Sudoscan^TM^ a useful device for assessing the presence of dysautonomia by evaluating the functionality of small nerve fibers in the skin.

Our study, however, has limitations, mostly due to the small sample of the population and the lack of a correlation with at least one other index of autonomic dysfunction in other systems (e.g., cardiovascular or respiratory). The sample size was limited to very co-operative subjects because it is necessary to maintain a very still position for a considerable amount of time in order to carry out the Sudoscan^TM^ recording. Such technical limitations have hampered the investigation of younger children and patients with intellectual disabilities, possibly associated with drug-resistant seizures. Indeed, we investigated only subjects with drug-sensitive epilepsies, suggesting that dysautonomic features may belong to the phenotypic spectrum of epilepsy, irrespective of the disease course. However, a comparison with drug-resistant patients would be strongly desirable, as well as a systematic exploration of neuroimaging features—especially white matter alterations and abnormalities in the morphometry of CAN regions. Likewise, a wider population could allow confirmation of our findings and also evaluation of differences in conductivity in specific age subgroups or according to the type of epilepsy (temporal vs. extra-temporal lobe epilepsies). The limitations also relate to a lack of correlation with other validated measures of dysautonomia, either objectively measured by the aforementioned instruments or derived from targeted questionnaires such as the Composite Autonomic Symptom Score (COMPASS) [53]. However, the reliability of Sudoscan^TM^ testing in unveiling peripheral dysautonomia appears robust for different chronic disorders with neurological involvement [54].

Further studies are therefore needed to clearly define the relationship between epilepsy and dysautonomia, especially in the pediatric age group, to investigate which are the most accurate methods to assess the associated symptoms, and to investigate whether dysautonomia may play a role on the overall clinical outcome.

## Figures and Tables

**Table 1:** Demographic and clinical characteristics of the study population.

Patient	Age (Years)	Sex	Epilepsy Type	Aetiology	Duration of Active Epilepsy (Years)	Drug Resistance	Current Seizure Frequency	Current ASMs	Cognitive Level	Brain MRI Findings
#1	17	M	Focal	unknown	3	no	seizure free	VPA	Normal	Normal
#2	20	F	Generalized	unknown	5	no	yearly	VPA	Bordeline	Normal
#3	21	M	Generalized	unknown	5	no	seizure free	-	n/a	n/a
#4	18	M	Focal	unknown	2	no	seizure free	-	Normal	Normal
#5	7	M	Unknown	unknown	1	no	seizure free	-	Normal	Left temporal lobe arachnoid cyst
#6	15	F	Generalized	unknown	1	no	seizure free	-	Normal	Normal
#7	17	M	Focal	unknown	7	no	seizure free	VPA	Normal	Normal
#8	7	F	Generalized	structural	1	no	seizure free	VPA	Normal	Poroencephaly
#9	9	F	Generalized	genetic (*SLC6A1*)	3	no	seizure free	VPA	Mild ID	Normal
#10	20	F	Generalized	unknown	1	no	seizure free	VPA	n/a	Normal
#11	5	M	Combined	unknown	3	no	yearly	VPA, ETS	Normal	Normal
#12	19	F	Generalized	unknown	9	no	seizure free	VPA	Normal	n/a
#13	15	M	Generalized	unknown	2	no	monthly	VPA, ETS	Moderate ID	Ventricular dysmorphism
#14	14	F	Focal	unknown	7	no	seizure free	CBZ	Normal	Normal
#15	24	F	Focal	structural	8	no	seizure free	VPA	n/a	Poroencephaly
#16	7	M	Generalized	unknown	2	no	seizure free	VPA	Normal	Normal
#17	8	F	Generalized	unknown	6	no	yearly	-	Normal	Normal
#18	15	F	Generalized	unknown	2	no	seizure free	-	n/a	Normal

## Data Availability

The original data presented in the study are openly available in Zenodo at https://zenodo.org/records/12071854 (accessed on 18 June 2024).

## Supplementary Materials

The following supporting information can be downloaded at: https://www.mdpi.com/article/10.3390/brainsci14070670/s1.

## Abbreviations

ASM—anti-seizure medications; ASN—autonomic nervous system; CAN—central autonomic network; CBZ—Carbamazepine; EDA—electrodermal activity; EEG—electroencephalogram; ESD—electrodermal skin conductivity; ETS—Ethosuximide; FDA—Food and Drug Administration; HRV—heart rate variability; MRI—magnetic resonance imaging; PGES—post-ictal generalized EEG suppression; PWE—people with epilepsy; SeLeAS—Self-Limited Epilepsy with Autonomic Seizures; SUDEP—sudden unexpected death in epilepsy; VNS—vagus nerve stimulation; VPA—Valproate.

## References

[1] Wehrwein E.A., Orer H.S., Barman S.M. (2016). Overview of the Anatomy, Physiology, and Pharmacology of the Autonomic Nervous System. Compr. Physiol..

[2] Serratrice J., Verschueren A., Serratrice G. (2013). Sistema Nervoso Autonomo. EMC Neurol..

[3] Goldstein D.S., Robertson D., Esler M., Straus S.E., Eisenhofer G. (2002). Dysautonomias: Clinical Disorders of the Autonomic Nervous System. Ann. Intern. Med..

[4] Axelrod F.B. (2013). Genetic Autonomic Disorders. Semin. Pediatr. Neurol..

[5] Axelrod F.B., Chelimsky G.G., Weese-Mayer D.E. (2006). Pediatric Autonomic Disorders. Pediatrics.

[6] Reichgott M.J., Walker H.K., Hall W.D., Hurst J.W. (1990). Clinical Evidence of Dysautonomia. Clinical Methods: The History, Physical, and Laboratory Examinations.

[7] Rafanelli M., Walsh K., Hamdan M.H., Buyan-Dent L. (2019). Autonomic Dysfunction: Diagnosis and Management. Handb. Clin. Neurol..

[8] Ming X., Bain J.M., Smith D., Brimacombe M., Gold von-Simson G., Axelrod F.B. (2011). Assessing Autonomic Dysfunction Symptoms in Children: A Pilot Study. J. Child Neurol..

[9] Hebson C.L., McConnell M.E., Hannon D.W. (2019). Pediatric Dysautonomia: Much-Maligned, Often Overmedicated, but Not as Complex as You Think. Congenit. Heart Dis..

[10] Specchio N., Wirrell E.C., Scheffer I.E., Nabbout R., Riney K., Samia P., Guerreiro M., Gwer S., Zuberi S.M., Wilmshurst J.M. (2022). International League Against Epilepsy Classification and Definition of Epilepsy Syndromes with Onset in Childhood: Position Paper by the ILAE Task Force on Nosology and Definitions. Epilepsia.

[11] Parikh S., Gupta A. (2013). Autonomic Dysfunction in Epilepsy and Mitochondrial Diseases. Semin. Pediatr. Neurol..

[12] Rodriguez-Quintana J., Bueno-Florez S., Mora-Muñoz L., Orrego-González E., Barragan A.M., Suárez-Burgos F., Velez-Van-Meerbeke A., Cendes F. (2023). Dysautonomia in People with Epilepsy: A Scoping Review. Seizure.

[13] Fisher R.S., Acevedo C., Arzimanoglou A., Bogacz A., Cross J.H., Elger C.E., Engel J., Forsgren L., French J.A., Glynn M. (2014). ILAE Official Report: A Practical Clinical Definition of Epilepsy. Epilepsia.

[14] Casellini C.M., Parson H.K., Richardson M.S., Nevoret M.L., Vinik A.I. (2013). Sudoscan, a Noninvasive Tool for Detecting Diabetic Small Fiber Neuropathy and Autonomic Dysfunction. Diabetes Technol. Ther..

[15] Gavan D.E., Gavan A., Bondor C.I., Florea B., Bowling F.L., Inceu G.V., Colobatiu L. (2022). SUDOSCAN, an Innovative, Simple and Non-Invasive Medical Device for Assessing Sudomotor Function. Sensors.

[16] EH Schwarz P., Brunswick P., Calvet J.-H. (2011). EZSCANTM a New Technology to Detect Diabetes Risk. Br. J. Diabetes Vasc. Dis..

[17] Berg A.T., Coffman K., Gaebler-Spira D. (2021). Dysautonomia and Functional Impairment in Rare Developmental and Epileptic Encephalopathies: The Other Nervous System. Dev. Med. Child Neurol..

[18] Thijs R.D., Ryvlin P., Surges R. (2021). Autonomic Manifestations of Epilepsy: Emerging Pathways to Sudden Death?. Nat. Rev. Neurol..

[19] Ryvlin P., Nashef L., Lhatoo S.D., Bateman L.M., Bird J., Bleasel A., Boon P., Crespel A., Dworetzky B.A., Høgenhaven H. (2013). Incidence and Mechanisms of Cardiorespiratory Arrests in Epilepsy Monitoring Units (MORTEMUS): A Retrospective Study. Lancet Neurol..

[20] Sahly A.N., Shevell M., Sadleir L.G., Myers K.A. (2022). SUDEP Risk and Autonomic Dysfunction in Genetic Epilepsies. Auton. Neurosci..

[21] Delogu A.B., Spinelli A., Battaglia D., Dravet C., De Nisco A., Saracino A., Romagnoli C., Lanza G.A., Crea F. (2011). Electrical and Autonomic Cardiac Function in Patients with Dravet Syndrome. Epilepsia.

[22] Marchal R., Rheims S. (2023). Assessing Epilepsy-Related Autonomic Manifestations: Beyond Cardiac and Respiratory Investigations. Neurophysiol. Clin. Clin. Neurophysiol..

[23] Labuz-Roszak B., Pierzchała K. (2009). Assessment of Autonomic Nervous System in Patients with Epilepsy in the Interictal State. A Pilot Study. Neurol. Neurochir. Pol..

[24] Casanovas Ortega M., Bruno E., Richardson M.P. (2022). Electrodermal Activity Response during Seizures: A Systematic Review and Meta-Analysis. Epilepsy Behav..

[25] Sarkis R.A., Thome-Souza S., Poh M.-Z., Llewellyn N., Klehm J., Madsen J.R., Picard R., Pennell P.B., Dworetzky B.A., Loddenkemper T. (2015). Autonomic Changes Following Generalized Tonic Clonic Seizures: An Analysis of Adult and Pediatric Patients with Epilepsy. Epilepsy Res..

[26] El Atrache R., Tamilia E., Amengual-Gual M., Mohammadpour Touserkani F., Yang Y., Wang X., Ufongene C., Sheehan T., Cantley S., Jackson M. (2021). Association between Semiologic, Autonomic, and Electrographic Seizure Characteristics in Children with Generalized Tonic-Clonic Seizures. Epilepsy Behav. EB.

[27] Rajakulendran S., Nashef L. (2015). Postictal Generalized EEG Suppression and SUDEP: A Review. J. Clin. Neurophysiol. Off. Publ. Am. Electroencephalogr. Soc..

[28] Poh M.-Z., Loddenkemper T., Reinsberger C., Swenson N.C., Goyal S., Madsen J.R., Picard R.W. (2012). Autonomic Changes with Seizures Correlate with Postictal EEG Suppression. Neurology.

[29] Horinouchi T., Sakurai K., Munekata N., Kurita T., Takeda Y., Kusumi I. (2019). Decreased Electrodermal Activity in Patients with Epilepsy. Epilepsy Behav. EB.

[30] Vieluf S., Reinsberger C., El Atrache R., Jackson M., Schubach S., Ufongene C., Loddenkemper T., Meisel C. (2020). Autonomic Nervous System Changes Detected with Peripheral Sensors in the Setting of Epileptic Seizures. Sci. Rep..

[31] Drake M.E., Andrews J.M., Castleberry C.M. (1998). Electrophysiologic Assessment of Autonomic Function in Epilepsy. Seizure.

[32] Koseoglu E., Kucuk S., Arman F., Ersoy A.O. (2009). Factors That Affect Interictal Cardiovascular Autonomic Dysfunction in Temporal Lobe Epilepsy: Role of Hippocampal Sclerosis. Epilepsy Behav. EB.

[33] Sivathamboo S., Perucca P. (2021). Interictal Autonomic Dysfunction. Curr. Opin. Neurol..

[34] Bordier L., Dolz M., Monteiro L., Névoret M.-L., Calvet J.-H., Bauduceau B. (2016). Accuracy of a Rapid and Non-Invasive Method for the Assessment of Small Fiber Neuropathy Based on Measurement of Electrochemical Skin Conductances. Front. Endocrinol..

[35] Vinik A.I., Smith A.G., Singleton J.R., Callaghan B., Freedman B.I., Tuomilehto J., Bordier L., Bauduceau B., Roche F. (2016). Normative Values for Electrochemical Skin Conductances and Impact of Ethnicity on Quantitative Assessment of Sudomotor Function. Diabetes Technol. Ther..

[36] Leclair-Visonneau L., Bosquet T., Magot A., Fayet G., Gras-Le Guen C., Hamel A., Péréon Y. (2016). Electrochemical Skin Conductance for Quantitative Assessment of Sweat Function: Normative Values in Children. Clin. Neurophysiol. Pract..

[37] Barot N., Nei M. (2019). Autonomic Aspects of Sudden Unexpected Death in Epilepsy (SUDEP). Clin. Auton. Res. Off. J. Clin. Auton. Res. Soc..

[38] Sevcencu C., Struijk J.J. (2010). Autonomic Alterations and Cardiac Changes in Epilepsy. Epilepsia.

[39] Liu H., Yang Z., Meng F., Guan Y., Ma Y., Liang S., Lin J., Pan L., Zhao M., Qu W. (2017). Impairment of Heart Rhythm Complexity in Patients with Drug-Resistant Epilepsy: An Assessment with Multiscale Entropy Analysis. Epilepsy Res..

[40] Dütsch M., Devinsky O., Doyle W., Marthol H., Hilz M.J. (2004). Cerebral Autoregulation Improves in Epilepsy Patients after Temporal Lobe Surgery. J. Neurol..

[41] Mukherjee S., Tripathi M., Chandra P.S., Yadav R., Choudhary N., Sagar R., Bhore R., Pandey R.M., Deepak K.K. (2009). Cardiovascular Autonomic Functions in Well-Controlled and Intractable Partial Epilepsies. Epilepsy Res..

[42] El-Rashidy O.F., Shatla R.H., Youssef O.I., Samir E. (2015). Cardiac Autonomic Balance in Children with Epilepsy: Value of Antiepileptic Drugs. Pediatr. Neurol..

[43] Isojärvi J.I., Ansakorpi H., Suominen K., Tolonen U., Repo M., Myllylä V.V. (1998). Interictal Cardiovascular Autonomic Responses in Patients with Epilepsy. Epilepsia.

[44] Mativo P., Anjum J., Pradhan C., Sathyaprabha T.N., Raju T.R., Satishchandra P. (2010). Study of Cardiac Autonomic Function in Drug-Naïve, Newly Diagnosed Epilepsy Patients. Epileptic Disord. Int. Epilepsy J. Videotape.

[45] Berilgen M.S., Sari T., Bulut S., Mungen B. (2004). Effects of Epilepsy on Autonomic Nervous System and Respiratory Function Tests. Epilepsy Behav. EB.

[46] Kasahara Y., Yoshida C., Nakanishi K., Fukase M., Suzuki A., Kimura Y. (2020). Alterations in the Autonomic Nerve Activities of Prenatal Autism Model Mice Treated with Valproic Acid at Different Developmental Stages. Sci. Rep..

[47] Galluzzi S., Nicosia F., Geroldi C., Alicandri A., Bonetti M., Romanelli G., Zulli R., Frisoni G.B. (2009). Cardiac Autonomic Dysfunction Is Associated with White Matter Lesions in Patients with Mild Cognitive Impairment. J. Gerontol. A. Biol. Sci. Med. Sci..

[48] Neuhaus E., Bitzer F., Held N.R., Bauer T., Gaubatz J., von Wrede R., Baumgartner T., Rácz A., Becker V., Surges R. (2024). Volumetric Gray Matter Findings in Autonomic Network Regions of People with Focal Epilepsy. J. Neuroimaging Off. J. Am. Soc. Neuroimaging.

[49] Ballerini A., Tondelli M., Talami F., Molinari M.A., Micalizzi E., Giovannini G., Turchi G., Malagoli M., Genovese M., Meletti S. (2022). Amygdala Subnuclear Volumes in Temporal Lobe Epilepsy with Hippocampal Sclerosis and in Non-Lesional Patients. Brain Commun..

[50] Sklerov M., Dayan E., Browner N. (2019). Functional Neuroimaging of the Central Autonomic Network: Recent Developments and Clinical Implications. Clin. Auton. Res. Off. J. Clin. Auton. Res. Soc..

[51] Bartolini E., Pesaresi I., Fabbri S., Cecchi P., Giorgi F.S., Sartucci F., Bonuccelli U., Cosottini M. (2014). Abnormal Response to Photic Stimulation in Juvenile Myoclonic Epilepsy: An EEG-fMRI Study. Epilepsia.

[52] Whelan C.D., Altmann A., Botía J.A., Jahanshad N., Hibar D.P., Absil J., Alhusaini S., Alvim M.K.M., Auvinen P., Bartolini E. (2018). Structural Brain Abnormalities in the Common Epilepsies Assessed in a Worldwide ENIGMA Study. Brain J. Neurol..

[53] Sletten D.M., Suarez G.A., Low P.A., Mandrekar J., Singer W. (2012). COMPASS 31: A Refined and Abbreviated Composite Autonomic Symptom Score. Mayo Clin. Proc..

[54] Moreno-Moraleda E., González-Moreno J., Cisneros-Barroso E., Ribot-Sansó M.A., Ripoll-Vera T., Descals C., Uson M., Montalà J.C., Figuerola A., Rodríguez A. (2024). Validating the Usefulness of Sudoscan in ATTRv: A Single Centre Experience. Neurol. Sci. Off. J. Ital. Neurol. Soc. Ital. Soc. Clin. Neurophysiol..

