# Supplementary material for: Children and Young Adults with Epilepsy Exhibit an Interictal Autonomic Dysfunction: A Prospective Exploratory Study
Source: Brain Sci. 2024 Jun 29;14(7):670. doi: 10.3390/brainsci14070670 (PMC11274926; doi:10.3390/brainsci14070670)
Supplement: Supplementary file 1 [file brainsci-14-00670-s001.zip › brainsci-3077828-supplementary.pdf]

SUPPLEMENTARY MATERIAL

Details of the therapeutic regimen of Patients on valproate treatment

**Patient**	**Duration of VPA treatment (years - yy, months - mm)**	**VPA dose at the time of registration (mg/die)**
**#1**	3 yy 0 mm	1200
**#2**	0 yy 8 mm	1000
**#8**	0 yy 3 mm	700
**#9**	0 yy 10 mm	600
**#10**	4 yy 0 mm	500
**#11**	0 yy 3 mm	1000
**#12**	3 yy 10 mm	450
**#13**	1 yy 3 mm	500
**#15**	10 yy 0 mm	750
**#16**	3 yy 4 mm	500

Median duration of VPA intake: 3 years 2 months (range 3 months-10 years)

Mean VPA dose at the time of registration: 720 mg ±262 mg
